# A 10-week intergenerational program bringing together community-living older adults and preschool children (INTERACTION): a pilot feasibility non-randomised clinical trial

**DOI:** 10.1186/s40814-024-01446-y

**Published:** 2024-02-21

**Authors:** Mei Ling Lim, Christine Zammit, Ebony Lewis, Nicole Ee, Genevieve Maiden, Micah Goldwater, Eva Kimonis, Gail Kenning, Kenneth Rockwood, Anneke Fitzgerald, Katrina Radford, Hiroko Dodge, Stephanie A. Ward, Kim Delbaere, Ruth Peters

**Affiliations:** 1 Ageing and Neurodegeneration, Neuroscience Research Australia, Sydney, Australia; 2 School of Population Health, University of New South Wales, Sydney, Australia; 3 School of Psychology, University of New South Wales, Sydney, Australia; 4 Ageing Futures Institute, University of New South Wales, Sydney, Australia; 5 War Memorial Hospital, Uniting, South Eastern Local Health District, Sydney, Australia; 6 Faculty of Science, University of Sydney, Sydney, Australia; 7 fEEL (felt Experience and Empathy Lab), University of New South Wales, Sydney, Australia; 8 Department of Medicine, Dalhousie University, Halifax, Canada; 9 Department of Business Strategy and Innovation, Griffith University, Nathan, Australia; 10 Department of Employment Relations and Human Resource Management, Griffith University, Nathan, Australia; 11 Massachusetts General Hospital, Harvard Medical School, Boston, MA USA; 12 Centre for Healthy Brain Ageing, School of Psychiatry, University of New South Wales, Sydney, Australia; 13 Falls, Balance and Injury Research Centre, Neuroscience Research Australia, Sydney, Australia; 14 Neurology, The George Institute for Global Health, Sydney, Australia

## Abstract

**Background:**

Social isolation and low levels of physical activity are strong drivers for frailty, which is linked to poor health outcomes and transition to long-term care. Frailty is multifactorial, and thus an integrated approach is needed to maintain older adults’ health and well-being. Intergenerational programs represent a novel multifactorial approach to target frailty, social isolation and physical decline but these have not yet been rigorously tested in Australia. Here, we present the results of our pilot study which aimed to test the feasibility of a 10-week intergenerational program between older adults and preschool children.

**Methods:**

A non-randomised wait-listed controlled trial was conducted. Participants were allocated to either the intervention or wait-list control group. The intervention group received 10 weekly 2-h intergenerational sessions led by trained child educators; the control group continued with their usual routine and received their intergenerational program after the 10-week control period. All participants were assessed at baseline and 10 weeks. The primary outcome was the feasibility and acceptability of the program including measures of recruitment eligibility, adherence and effective data collection across the multiple domains important for frailty, including functional mobility and balance, grip strength, cognitive function, mood, social engagement, quality of life and concerns about falling.

**Results:**

Nineteen adults were included, with nine in the intervention and ten in the control group. A total of 42% of older adults screened were eligible, 75% of participants were present at each intervention session and the overall attrition rate was 21% (*n* = 4). The reasons for participant absence were primarily health-related. Missing data was minimal for the majority of assessments but more apparent for the cognitive testing where completion rates ranged from 53 to 79% for baseline tests and 73 to 100% for those who received follow-up testing.

**Conclusions:**

The high program compliance and low attrition show that a 10-week intergenerational program embedded in the local community, designed for community-living older adults and preschool children, is feasible and acceptable to older adults. Our next trial will test the efficacy of intergenerational programs in this setting.

**Supplementary Information:**

The online version contains supplementary material available at 10.1186/s40814-024-01446-y.

## Key messages regarding feasibility

- Uncertainties regarding feasibility: There are limited robust studies on the short-term and long-term benefits of intergenerational programs for community-living older adults, as opposed to studies conducted in aged care facilities such as nursing homes, and the best practice to deliver such intergenerational programs.
- Key feasibility findings: Our study shows that a 10-week wait-listed intergenerational program with community-living older adults and preschool children in a community space attached to a preschool led by two preschool educators is feasible. We also found that the cognitive tests were too long and the timing of the follow-up assessments could have affected the self-reported outcomes.
- Implications of the findings to design of the main study: The feasibility study shows that shorter cognitive tests would be more desirable, and assessing participants just before the completion of the intergenerational sessions so that the self-reported outcomes are not affected by the completion of the sessions.

## Background

Social isolation in older adults is a global publicAQ health concern [1]. Older adults who are well-embedded in the community and have good social relationships tend to have better health outcomes, including better cognitive and physical function and are less likely to become frail [2–5]. High levels of social isolation have been associated with increased mortality, reduced functional status, lower everyday physical activity and greater sedentary time [6, 7]. More importantly, high levels of social isolation are associated with higher risks of developing dementia or cognitive decline in older people [8, 9].

Social isolation and low levels of physical activity in older adults are strong drivers for frailty, with frailty linked to poor health outcomes and increased risk of hospitalisation and transition to long-term care [10–14]. Yet, maintaining reliable positive social interactions and adequate physical activity in later life can be challenging due to age-related constraints such as poor health and mobility, driving cessation and living alone. Naturalistic, community-embedded solutions, such as intergenerational programs that provide opportunities for older adults to build positive social relationships and increase physical activity and cognitive engagement, might reduce the risk of unwanted health consequences, including frailty.

Intergenerational programs that bring non-familial older adults and children together to engage in purposeful joint activities for mutual benefit have shown potential gains for older adults in self-esteem, self-worth, perceived usefulness and reduced loneliness and anxiety [15]. Intergenerational programs often include activities that result in teaching and learning between the two generations. For example, teaching traditional songs or activities to children and supporting children to read or learn [16–18]. However, robust studies on the short-term and long-term benefits of intergenerational programs for community-living older adults, as opposed to studies conducted in aged care facilities such as nursing homes, remain limited. Few studies have utilised controlled and or randomised and non-randomised trial designs to test the effects of intergenerational activities on community-living older adults and children [19–24]. Likewise, a recent systematic review highlighted the need for more rigorous measurements in longitudinal studies with larger sample sizes to better understand best practices in delivering such intergenerational programs [15]. An explorative survey with community-living older adults and parents of preschool children also revealed a demand for community-embedded intergenerational programs and the need for an evidence-based framework to deliver the programs in a sustainable and effective way [25].

Intergenerational programs represent a multi-domain intervention that could have a pervasive impact on the physical, psychological, social, and cognitive health of community-living older adults. Multi-domain intergenerational programs often include physical activities combined with cognitive or relationship-building tasks to improve multiple health outcomes in older adults. Recent systematic and scoping reviews have consistently agreed that intergenerational programs have the potential to improve older adults’ physical and mental health, cognition, quality of life and social connectedness [15, 26–28]. However, more robust evidence with relevant outomes are needed. Here, we conduct a pilot study to test the feasibility and acceptability of a 10-week intergenerational program designed for community-living older adults and preschool children as measured by screen failure rates and intervention adherence. The secondary aims of this trial were to test the feasibility (completeness) of data collection across multiple domains including cognition, affect, quality of life, social network, physical performance and concerns about falling. We report only the results from the adult participants.

## Methods

### Study design

The Intergenerational Clinical Trial In at-risk Older adults and pre-school childreN (INTERACTION) is a non-randomised, wait-listed controlled trial conducted between January 2022 and September 2022. This was a 10-week trial of intergenerational practice at one site (a community-based preschool with an attached hall) compared to a contemporaneous wait-list control at another site (a community-based preschool with an attached hall). Both preschools were situated in the suburbs of Sydney, New South Wales (NSW), Australia, with similar geographical and sociodemographic characteristics. The wait-list control site received 10 weeks of the intergenerational program after their assessment (control) period ended. This clinical trial was prospectively registered with the Australian New Zealand Clinical Trials Registry (ACTRN12622000368730), and ethical approval was obtained from the University of New South Wales Human Research Ethics Committee (HC210975). Written informed consent was obtained from all participants before the screening assessment. The reporting of this trial follows the Consolidated Standards of Reporting Trials (CONSORT) statement extension to randomised pilot and feasibility trial recommendations [29].

### Participant and recruitment

Participants were recruited from the community through online advertisements via local Facebook Groups, flyers distributed in local libraries, churches and shops and referrals from aged care providers and community organisations. Participants were included if they were aged 65 or over, had a baseline Montreal Cognitive Assessment (MOCA) [30] score of 22 or more, lived in the community, were able to travel to the intervention site, and were able to sit and stand from a chair with arms, were able to walk 6 m with or without a walking aid, spoke English, were fully-vaccinated against COVID-19 and had a valid Working With Children Check (WWCC) (required by law in NSW before voluntary activity or work with children) [31]. Exclusion criteria included people with speech or sensory deficits that prevent interaction and who did not have a valid WWCC. The child participants and their results are not discussed in detail here, but briefly, they were aged 3–4 and attended the participating preschool sites. Written informed consent was sought from the child’s parents and assent was sought from the participating children.

### Procedure

Older adults interested in joining the program provided their details to the research team. During an initial phone call to interested participants, the research team member explained the study procedures and checked their availability for the intervention period. Participants who were still interested were then invited to an in-person appointment at the participating sites or Neuroscience Research Australia (a medical research institute with suitable assessment rooms) for their screening assessments. Written informed consent was sought from all interested participants prior to screening. After screening, eligible participants were invited to continue with the baseline assessments on the same day. Participants were allocated to either the intervention or control group based on their proximity to the participating sites. After the 10-week intervention period, all participants were invited back for a follow-up assessment.

### Intervention

The intervention consisted of 10 weekly 2-h sessions delivered in a hall adjacent to the preschool site. The sessions occurred at the same time each week and included multi-modal intergenerational activities targeting physical, cognitive function and mood and were tailored to fit with the selected Early-Years Learning Framework [32] (followed by Australian preschools) (see appendix 1). Sessions were led and delivered by two experienced child educators working at the preschool as they were most familiar with the child participants. The educators were also supported by the research team with information on how to conduct intergenerational activities and the aims of the study. Additional support to help set up the session space before, during and after the intergeneration sessions was provided by two research staff or one research staff member and a trained volunteer experienced in working with older adults. The weekly program was planned together by the researchers and the two educators leading the sessions. Each weekly session was guided by one main theme (e.g. construction, gardening, animals) and included tasks targeting physical activity, cognitive stimulation and social interaction (see Appendix 1 and 2 for an example of the program). All tasks required adult and child participants to work in groups or pairs. At the end of each session, the older adults were given the opportunity to provide feedback on which aspects of the session they enjoyed or disliked. A weekly post-session debrief was also conducted to allow the educators and the research team members to reflect on the strengths and areas for improvement of the session, as well as to plan activities for the following week.

### Control

**Fig. 1:**
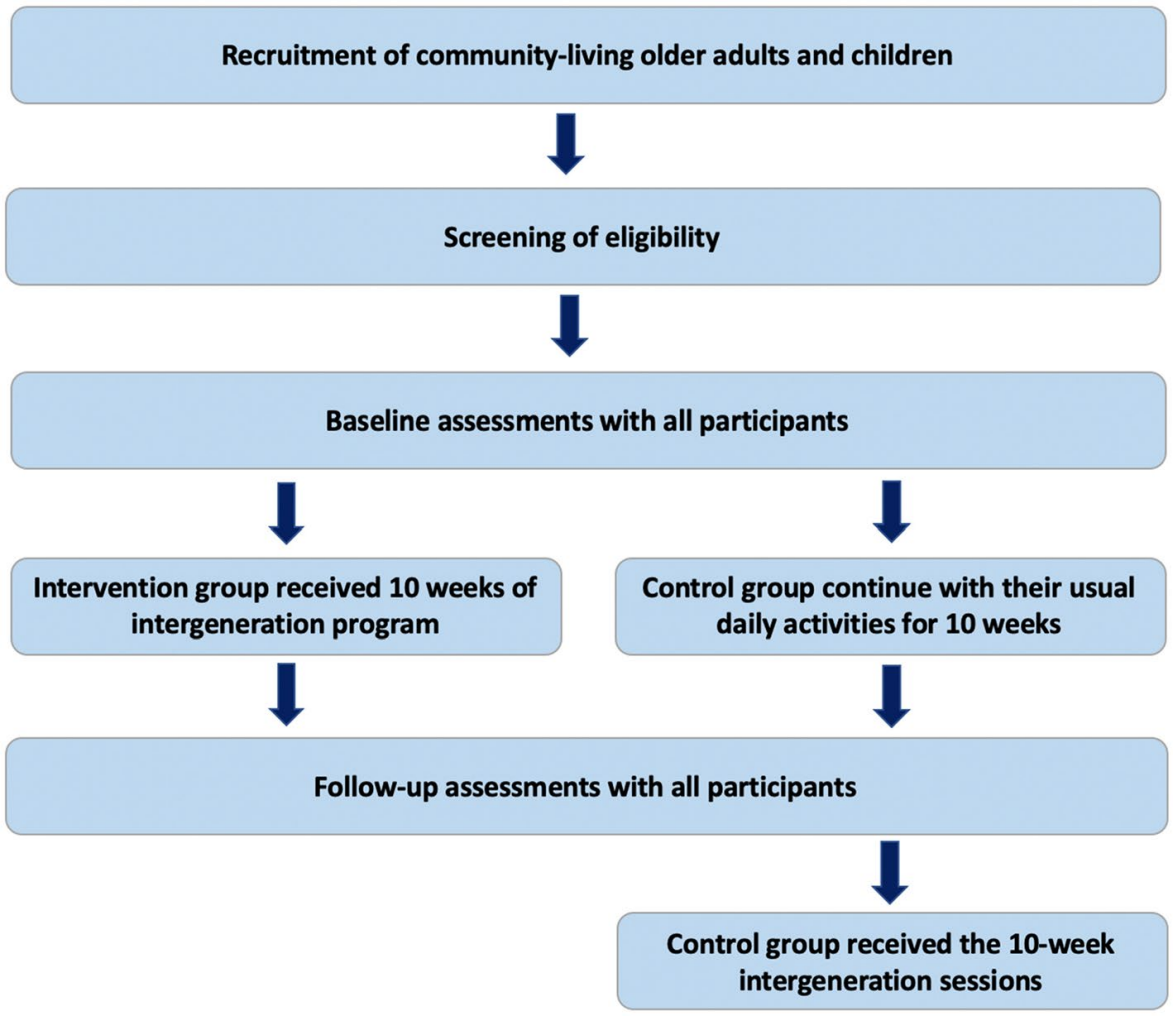
Process flow of trial

The control group received no intergenerational practice sessions during the intervention period and were asked to continue their day-to-day activities. The control group received the 10-week intergenerational sessions after completing their follow-up assessments (see Fig. 1).

### Outcome measures

The primary outcome was the feasibility and acceptability of the intergenerational program. Feasibility and acceptability were measured by (i) the number of participants screened, eligible and included in the study, reasons for ineligibility (ii) attendance of participants (older adults) at intervention sessions and reasons for lack of attendance. Attendance and reasons for absence were recorded by the researcher on-site for each intergenerational session.

The secondary outcomes were to test the feasibility of data collection, measured by the completeness of data at baseline and follow-up, across a number of domains as required for the assessment of frailty measured using an index of deficits (the Frailty Index (FI)). The domains included cognition, affect, quality of life, social network, physical performance and concerns about falling. The research team administered all physical and cognitive tests. Grip strength was assessed using a Jamar dynamometer [33]. Functional mobility and balance were assessed using the Short physical performance battery (SPPB) test, [34] and the Timed up and go (TUG) [35]. Cognitive function was measured with the validated and widely used computerised Cambridge Neuropsychological Test Automated Battery (CANTAB®) [36, 37]. Executive function was assessed using three tests from the CANTAB®, the multitasking test, stockings of Cambridge, and spatial working memory tests. Attention was assessed using two tests from CANTAB®, the rapid visualisation information processing (RVPA) and reaction time tests. All participants completed a practice test (i.e. the motor screening task) before the actual cognitive tests.

The remaining psychological and social measures were self-reported using questionnaires. Mood was assessed using the Positive And Negative Affect Scale (PANAS) [38]. Quality of life was assessed using the 36-item short form health survey (SF-36) [39]. Social support was assessed using the Lubben Social Network Scale-6 (LSNS-6) [40]. Concerns about falling were measured with the 30-item Iconographical Falls Efficacy Scale (IconFES) [41]. The presence or absence of 10 commonly occurring clinical conditions relevant to ageing was also collected with a self-report questionnaire. At baseline, demographic and general health information including age, gender, education, number of years living in Australia and place of birth. A telephone-administered Fatigue, Resistance, Ambulation, Illness and Loss (FRAIL) Scale [42] assessed phenotypic frailty and prefrailty. Completeness of data collection was also measured to provide an understanding of areas where older adults might find question completion onerous.

Process outcomes were also assessed at the end of the 10-week intervention. Participants from the intervention group were asked five questions about their program experience. Participants were asked to rate how much they liked the overall program, the activities, the program duration and their interactions with the children and adult participants on a scale ranging from 1 (I didn’t like it at all) to 10 (I liked it a lot).

### Sample size

Since this was a feasibility trial, a formal sample size calculation was not required; however, based on our prior work, for our primary outcome of the proportion of participants screened to those found eligible and included in the study, it was estimated that 80 needed to be screened with 1/3 eligible, leading to approximately 24 adult participants [43]. We further anticipated that up to four older adults would be lost to follow-up during the intervention period; thus, 20 would complete the 10-week intervention. The progression criterion for our feasibility trial to the larger efficacy trial was based on achieving a successful adult participant screening/eligible rate.

### Analyses

Statistical analyses were performed using SPSS version 26 and SAS v9.3. Due to the nature of the study, descriptive statistics were used to describe feasibility-based outcomes including recruitment, retention and completeness of data collection measures. Completeness of data will be presented as the percentage of completed data provided by (i) all participants at baseline and (ii) remaining participants at follow-up assessments.

## Results

### Feasibility

**Fig. 2:**
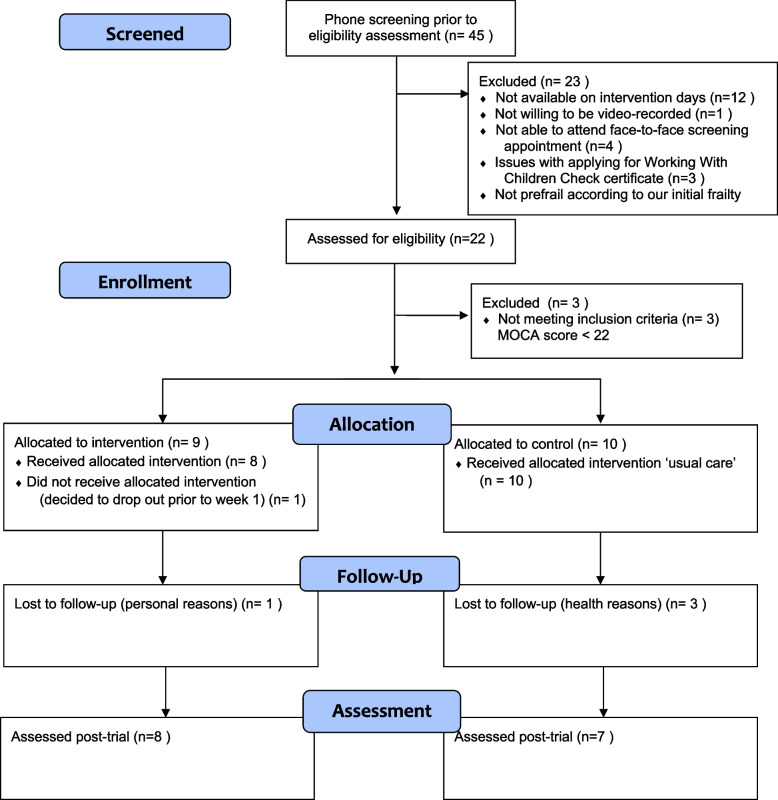
Participants flow diagram

Enrolment procedures were conducted in accordance with the CONSORT guidelines and are presented in Fig. 2. A total of 45 older adults expressed interest in this trial. After the initial phone call, 23 did not proceed to the face-to-face screening assessments for a range of reasons, including being unavailable to attend the in-person screening appointments or the pre-determined intergenerational intervention sessions held during the school term, unwillingness to be photographed or video-recoded during the intergenerational sessions, having issues with obtaining the WWCC certificate due to missing required documents and not meeting an initial screening requirement of being “prefrail” (*n* = 3). The initial requirement for the older adults to have a score of 1 to 2, which indicates prefrailty, on a telephone-administered Fatigue, Resistance, Ambulation, Illness and Loss (FRAIL) Scale [42] was removed partway through recruitment when it became clear, due to inconsistencies in the information provided by the participants during the screening call, that it was subjectively influenced by participant perception of what might be required to enter the trial. Twenty-two older adults attended the in-person screening assessment, and three adults were excluded as their MOCA score was less than 22. Nineteen older adults were included in total. Nine participants were in the intervention group and ten in the control group. The attrition rate was 21% (*n* = 4) at 10 weeks (three among the control group and one among the experimental group). One participant from the intervention group withdrew from the study prior to the start of the intervention (see Fig. 2). Participants who withdrew after consent had higher scores on the SPPB and were less frail compared to participants who remained in the trial.

Session attendance rates and reasons for absence for the intervention group are presented in Table 1. Nine out of the ten intergenerational sessions were conducted. Week 4 of the program was cancelled due to positive cases of COVID-19 among the child participants. All sessions had at least six out of eight participants present. Participants were absent from the sessions for a range of reasons, including poor sleep, going for day surgery, pain in the leg and COVID-19 or flu. One participant was absent from the first 2 weeks of the program as her WWCC was not approved till the third week of the intervention.

**Table 1:** Session attendance of the intervention group participants (*n* = 8) and reasons for absence

**Week**	**Number of participants present *n* (%)**	**Number of participants absent *n* (%)**	**Reasons for absence**
1	7 (87.5%)	1 (12.5%)	Awaiting WWCC approval
2	7 (87.5%)	1 (12.5%)	Awaiting WWCC approval
3	7 (87.5%)	7 (87.5%)	Medical reason (day surgery)
4	Session cancelled due to positive COVID cases among the children participants		
5	6 (75%)	2 (25%)	Medical reasons (leg pain, COVID)
6	6 (75%)	2 (25%)	Medical reason (flu), poor sleep
7	8 (100%)	0	
8	7 (87.5%)	1 (12.5%)	Medical reason (flu)
9	7 (87.5%)	1 (12.5%)	Poor sleep
10	8 (100%)	0	

### Baseline characteristics

The baseline characteristics of all participants are presented in Table 2. Most participants were women in their 70s, with high but non-perfect MoCA scores; about half were robust by the Frail Scale questionnaire (Table 2). There were no statistically significant differences between the intervention and control group on most baseline characteristics except the SPPB, TUG and iconFES scores, with the control group scoring better on these three tests as compared to the intervention group.

**Table 2:** Characteristics of all participants at baseline

**Characteristics**	**Total (*n* = 19)**	**Intervention group (*n* = 9)**	**Control group (*n* = 10)**
Age, years, mean (SD)	76.2 (6.4)	77.2 (5.1)	75.2 (7.6)
Female gender, *n* (%)	17 (89.5)	9 (100)	8 (80.0)
Years of education	14.00 [8.00]	12.00 [4.50]	16.00 [6.75]
Years in Australia	66.7 (15.5)	67.89 (13.0)	64.90 (15.9)
Country of birth *n* (%)			
Australia	12 (63.2)	5 (55.6)	7 (70.0)
Other countries	7 (36.8)	4 (44.4)	3 (30.0)
Number of comorbidities	2.00 [3.00]	3.00 [2.50]	2.00 [3.25]
MoCA (score)	25.9 (2.2)	27.1 (2.4)	26.0 (2.3)
Frail scale, *n* (%)			
Robust	10 (52.6)	4 (44.4)	6 (60.0)
Prefrail/frail	9 (47.4)	5 (55.6)	4 (40.0)
SPPB—lower extremity function (score)	10.00 [5.00]	6.00 [5.50]	11.00 [3.50]
Time up and Go (TUG) (seconds)	9.33 [4.19]	10.46 [6.45]	7.29 [3.59]
Grip strength (kilograms)	15.82 (8.04)	13.85 (5.30)	18.00 (8.24)
Near tandem balance (seconds)	30.00 [6.00]	30.00 [3.33]	30.00 [19.50]
IconFES concerns about falling (score)	55.6 (17.5)	64.00 (17.37)	48.10 (14.47)
PANAS negative affect* (score)	15.00 [9.00]	16.00 [9.50]	13.50 [10.50]
PANAS positive affect (score)	36.11 (7.45)	35.11 (5.30)	37.00 (9.18)
LSNS-6—social engagement (score)	17.42 (5.11)	17.44 (6.19)	17.40 (4.27]
SF36—general health (score)	65.26 (21.82)	63.33 (23.18)	67.00 (21.63)
SF36—physical function (score)	67.31 (26.84)	65.99 (29.23)	68.50 (26.04]
SF36—social function (score)	87.50 [38.00]	87.50 [43.75]	87.50 [37.50]
SF36—emotional wellbeing (score)	69.89 (10.69]	65.78 (12.35)	73.60 (7.82)
SF36—energy/fatigue (score)	57.37 (14.56)	53.33 (15.00)	61.00 (13.90)
SF36—pain* (score)	77.50 [35.00]	77.50 [40.00]	78.75 [33.12]
SF36—role limitations due to physical health (score)	100.00 [100.00]	100.00 [100.00]	50.00 [100.00]
SF36—role limitations due to emotional problems (score)	100.00 [33.00]	100.00 [50.00]	100.00 [33.33]

Note: Measurements in median and interquartile range represented in “[ ]” unless otherwise indicated

*Abbreviations*: *MoCA* Montreal Cognitive Assessment, *SPPB* Short Physical Performance Battery, *PANAS* Positive and Negative Affect Schedule, *LSNS* Lubben Social Network Scale, *SF36* 36-Item Short Form Health Survey, *IconFES* Iconographical Falls Efficacy Scale

*Higher scores = less favourable

### Completeness of data for secondary outcomes

At baseline, missing data were present in cognition and physical performance domains. *Physical performance – *Only 68% of the participants completed the Tandem balance test. *Cognition –* Missing data was present in all cognitive tests at baseline. The lowest completion rate was observed in the rapid visualisation information processing test (53%), followed by the Cambridge of Stocking test (68%). Higher levels of completion were recorded for the reaction time test (79%), multitasking test (79%) and spatial working memory test (74%). At the follow-up assessment, missing data (in those who completed the study) was only observed in two cognitive tests. A higher completion rate, as compared to the baseline, was seen in the rapid visualisation information processing test (73%), and reaction time test (93%). Anecdotally, during the in-person assessments participants mentioned that cognitive tests were too long and arduous (see Table 3).

**Table 3:** Mean and standard deviations of pre- and post-intervention scores (secondary outcomes) and the number of participants who completed each test/questionnaire

**Variables (secondary outcomes)**	**Intervention**				**Control**				**N(%) who completed the test**	
	**Baseline (*n* = 9)**	***n***	**10 week (*n* = 8)**	***n***	**Baseline (*n* = 10)**	**n**	**10 week (*n* = 7)**	***n***	**Baseline (*n* = 19)**	**10 week (*n* = 15)**
Number of comorbidities	2.75 ± 1.49	9	3.38 ± 1.51	8	2.86 ± 2.12	10	3.43 ± 2.37	7	19 (100)	15 (100)
SPPB—lower extremity function (score)	6.63 ± 2.83	9	7.50 ± 2.51	8	9.57 ± 1.99	10	9.86 ± 2.55	7	19 (100)	15 (100)
Near tandem balance test—balance ^a^ (seconds)	30.0 ±0.00	3	30.0 ±0.00	8	26.1 ± 10.2	10	23.1 ± 11.7	7	13 (68)	15 (100)
Time up and Go (TUG) (seconds)	12.33 ± 4.20	9	11.85 ± 4.80	8	9.93 ± 4.52	10	9.62 ± 5.15	7	19 (100)	15 (100)
Grip strength (kilograms)	14.5 ± 5.3	9	16.5 ± 5.6	8	15.9 ± 8.9	10	19.4 ± 8.1	7	19 (100)	15 (100)
IconFES—concerns about falling (score)	65.5 ± 17.9	9	63.9 ± 23.5	8	51.9 ± 15.0	10	55.3 ± 17.5	7	19 (100)	15 (100)
PANAS—mood negative affect (score)^a^	16.75 ± 4.98	9	15.63 ± 4.63	8	20.00 ± 9.00	10	16.00 ± 6.11	7	19 (100)	15 (100)
PANAS—mood positive affect (score)	33.75 ± 3.62	9	33.13 ± 4.05	8	35.43 ± 10.41	10	39.00 ± 9.09	7	19 (100)	15 (100)
LSNS-6—social engagement (score)	16.75 ± 6.22	9	15.38 ± 4.21	8	16.29 ± 3.50	10	14.86 ± 2.19	7	19 (100)	15 (100)
SF36 domains (score)										
Physical function	69.86 ± 28.68	9	66.39 ± 29.94	8	61.43 ± 25.77	10	59.21 ± 34.73	7	19 (100)	15 (100)
Social function	73.44 ± 27.90	9	65.63 ± 16.02	8	71.43 ± 26.72	10	89.29 ± 19.67	7	19 (100)	15 (100)
Emotional wellbeing	65.50 ± 13.17	9	72.5 ± 8.67	8	71.43 ± 8.46	10	74.29 ± 8.90	7	19 (100)	15 (100)
Energy/fatigue	52.50 ± 15.81	9	54.38 ± 17.41	8	58.57 ± 15.20	10	60.71 ± 24.05	7	19 (100)	15 (100)
Pain	65.63 ± 27.54	9	64.38 ± 22.27	8	67.86 ± 32.48.	10	74.29 ± 24.78	7	19 (100)	15 (100)
Role limitations due to physical health	62.50 ± 51.76	9	37.50 ± 46.29	8	28.57 ± 48.80	10	64.29 ± 40.56	7	19 (100)	15 (100)
Role limitations due to emotional problems	75.00 ± 38.83	9	45.83 ± 50.20	8	76.19 ± 37.09	10	95.24 ± 12.60	7	19 (100)	15 (100)
General Health	61.25 ± 23.87	9	57.50 ± 17.53	8	62.14 ± 22.15	10	62.86 ± 31.07	7	19 (100)	15 (100)
*Attention*										
Reaction time RTIMDMT* (ms)	432.93 ± 80.17	8	402.79 ± 128.30	7	297.21 ± 22.50	7	300.5 ± 52.50	7	15 (79)	14 (93)
Reaction time RTIMDRT* (ms)	487.00 ± 59.0	8	483.86 ± 39.78	7	458.36 ± 42.42	7	434.43 ± 60.69	7	15 (79)	14 (93)
Rapid visual info process RVPA	0.87 ± 0.05	6	0.85 ± 0.06	6	0.89 ± 0.03	4	0.91 ± 0.06	5	10 (53)	11 (73)
Rapid visual info process RVPMDL* (ms)	543.67 ± 136.41	6	606.67 ± 233.70	6	463.00 ± 20.50	4	445.50 ± 63.74	5	10 (53)	11 (73)
Rapid visual info process RVPPFA*	0.05 ± 0.12	7	0.02 ± 0.02	7	0.01 ± 0.01	5	0.01 ± 0.01	5	12 (63)	12 (80)
*Executive function*										
Multitasking MTTLMD* (ms)	761.75 ± 178.67	8	776.75 ± 127.21	8	779.07 ± 119.56	7	801.50 ± 173.51	7	15 (79)	15 (100)
Multitasking MTTTIC*	18.63 ± 10.94	8	20.50 ± 13.48	8	23.00 ± 21.36	7	26.00 ± 29.74	7	15 (79)	15 (100)
Stockings of Cambridge OTSMDLFC^ (ms)	20349.38± 8126.40	8	15161.25± 5817.18	8	13575.40 ± 4915.55	5	12631.60 ± 6926.21	7	13 (68)	15 (100)
Stockings of Cambridge OTSPSFC	6.38 ± 3.50	8	7.38 ± 3.74	8	6.60 ± 5.60	5	8.20 ± 5.59	7	13 (68)	15 (100)
Spatial working memory SWMBE468*	22.86 ± 7.67	7	23.14 ± 4.06	8	23.14 ± 9.42	7	19.57 ± 11.69	7	14 (74)	15 (100)
Spatial working memory SWMS*	9.71 ± 1.11	7	9.57 ± 1.81	8	9.71 ± 1.60	7	9.71 ± 1.98	7	14 (74)	15 (100)

All data are presented in mean ± standard deviation; ^*^higher scores indicate worse (“-ve”) performance; ^^^direction of score interpretation is complex; ^a^lower scores representing lower levels of negative affect

*ms* milliseconds, *N* number of participants who completed the questionnaire/test

### Process outcomes

The overall program experience of the intervention group participants was generally positive. The participants rated a score of 9.5 (interquartile range [IQR] = 1) for the overall program, 9.5 (IQR = 1.75) for the activities, 9 (IQR = 1.75) for the program length, 10 (IQR = 0) for the interactions with the children and 10 (IQR = 1) for the interactions with the adult participants.

## Discussion

The aim of this study was to evaluate the feasibility of an intergenerational program in community-living older adults and preschool children and thus generate learning to help design a future larger efficacy trial. Our study shows that running a 10-week wait-listed intergenerational program with community-living older adults and preschool children in a community space attached to a preschool led by two preschool educators is feasible. Overall, our intervention group participants showed a good attendance rate throughout the nine sessions, and all eight participants who started the program completed the 10-week program with no attrition at the end of 10 weeks. In contrast, there was a higher attrition rate in the wait-list control group than in the intervention group after 10 weeks. The absence of contact with the control group participants during the 10-week “wait list” period could thus be one of the reasons for the higher attrition in this group. Regular brief check-ins with the control group participants to keep them engaged while waiting for their “intervention” could possibly mitigate loss in future randomised wait-listed trials.

### Recruitment

The INTERACTION pilot trial highlights that recruiting older adults from local communities and aged care providers to participate in community programs, such as intergenerational practice, is feasible. However, it should be noted that we had to remove our participant selection criteria of being “pre-frail” at the initial stage (first 3 weeks) of our recruitment period. Initially, potential participants were screened for being “prefrail” over the phone using the FRAIL scale. As the FRAIL scale is a short 5-item, self-reported questionnaire, there may be a tendency for older adults to provide what they consider to be a more socially desirable answer for participation in the trial rather than to give a truthful answer around the sensitive topic of their health and functional status [44]. Therefore, it was challenging to identify pre-frail older adults since potential participants might score themselves as robust instead. Expanding the selection criteria further represents a pragmatic approach for future translational work and enabled us to recruit a more representative sample of the older adults living in the local community. The change in our recruitment criteria did not result in any difference in the baseline characteristics of the intervention and control group as recruitment of all participants happened during the same time period.

### Outcome measures

Levels of missing data were low for most secondary outcomes, except for the cognitive domain, and anecdotally during the in-person assessments participants mentioned that cognitive tests were too long and arduous. A shorter online cognitive testing battery may be more desirable in the next trial. Additionally, the outcome measures selected for this trial may not be the most meaningful outcome for our participants. Individualised outcome measures, such as the Goal Attainment Scaling (GAS) [45] may be a more relevant, scalable, and person-centered measure for future studies. Besides this, goal setting is a common behaviour change technique used in healthy ageing interventions and might further motivate the participants to be more engaged with the intergenerational program and provide a sense of purpose [46, 47]. Future intergenerational trials should consider including personal goal settings for adult participants to increase motivation and adherence to the program.

### Timing of the assessments

Our follow-up assessments occurred 1 week after the last session of the program. Anecdotally, many of the older adult participants expressed disappointment that the program had ended during their follow-up assessments with the researchers. It is possible that the intervention group participants were experiencing “a sense of loss” during the follow-up assessment period as the intergenerational program had just ended. This could potentially affect their self-reported outcomes indirectly when completing the follow-up assessments. Future intergenerational (wait-listed) studies should consider assessing participants just before the completion of the intergenerational sessions so that the self-reported outcomes are not affected indirectly because of the completion of the sessions.

## Strengths and limitations

This is the first non-randomised trial to explore the feasibility of a 10-week intergenerational program in community-living older adults and preschool children. However, we would like to acknowledge a few limitations. First, whilst our study included community-living older adults with a range of frailty levels, we were not able to include a fully representative sample of the older adult population. We excluded participants living in residential aged-care facilities who are often more frail than the community-living older adults. Further work needs to be carried out to examine the potential for such studies in these at-risk populations. Second, as this is a pilot study, our sample size was small and intentionally not powered to show an effect for the secondary outcomes, the characteristics of the older adults recruited at one site were different to those recruited at the other; however, we acknowledge that other methods can be applied to estimate the sample size required to establish feasibility for pilot studies [48]. Lastly, our pilot trial was limited to two preschools in Sydney’s more affluent suburbs. Our next large-scale clinical trial (ACTRN12623000127606) aims to include a wider selection of sites from different areas to support the generalisability of the results.

Future trials may also benefit from investigating specific targeted activities for improving physical and cognitive health or running for a longer duration (e.g. increasing the number of weeks) to refine interventions and target a particular effect on the health outcomes of the community-living older adults. For example, evidence has shown that combining physical and cognitive training delivered simultaneously in a dual-task format is efficacious in promoting physical and cognitive health in older adults with and without mild cognitive impairment [49, 50]. Therefore, adding more dual-task activities to the intergenerational program could potentially boost the effects of the intervention. Our intervention was also limited to 2 hours of intergenerational activities each week following our earlier scoping work where longer or more frequent sessions were reported as too tiring or inconvenient for older adult participants. Nevertheless, it is recommended for older adults to continue staying active beyond the 2 hours of intergenerational sessions, with at least 150 min of moderate-intensity aerobic physical activity a week to benefit their overall health, including physical function, cognitive outcomes and mental health [51]. Perhaps adding an education component to emphasise the importance of staying active for maintaining good physical function and cognitive health might be useful in getting older adults to stay physically active beyond the intergenerational sessions. Additionally, engaging and empowering older adults to be more actively involved in teaching the children, such as through storytelling or reading, might be beneficial to their cognitive function [18]. Furthermore, studies that add qualitative measures such as observations and video ethnographies may be important for small trials alongside quantitative measures. Going forwards, the next step is a larger efficacy trial with a randomised wait-listed controlled design to balance the participant characteristics in both groups. The INTERACTION trial extends the literature by demonstrating the feasibility for a future trial and identifying that the community is interested in intergenerational programs to bolster their health and well-being.

## Conclusion

This study shows that delivering a 10-week intergenerational program embedded in the local community, designed for community-living older adults and preschool children is feasible and acceptable to older adult participants. Our next trial will test the efficacy of intergenerational programs in this setting.

### Supplementary Information

**Additional file 1: Appendix 1.**

**Additional file 2: Appendix 2.** Sample INTERACTION intervention programs.

## Data Availability

Not available.
