# Supplementary material for: A 10-week intergenerational program bringing together community-living older adults and preschool children (INTERACTION): a pilot feasibility non-randomised clinical trial
Source: Pilot Feasibility Stud. 2024 Feb 21;10:37. doi: 10.1186/s40814-024-01446-y (PMC10880214; doi:10.1186/s40814-024-01446-y)
Supplement: Supplementary file 1 — Additional file 1: Appendix 1. [file 40814_2024_1446_MOESM1_ESM.docx]

**Appendix 1**

- EYLF Outcome 1.4: Children have a strong sense of identity: Children learn to interact in relation to others with care, empathy and respect

- EYLF Outcome 3.1: Children have a strong sense of wellbeing: Children become strong in their social, emotional and mental wellbeing

- EYLF Outcome 4.1: Children and confident and involved learners: Children develop a growth mindset and learning dispositions such as curiosity, cooperation, confidence, creativity, enthusiasm, persistence, imagination and reflexivity

- EYLF Outcome 4.4: Children are confident and involved learners: Children resource their own learning through connecting with people, place, technologies, and natural and processed materials

- EYLF Outcome 5.1: Children are effective communicators: Children interact verbally and non-verbally with others for a range of purposes.
