# Supplementary material for: A 10-week intergenerational program bringing together community-living older adults and preschool children (INTERACTION): a pilot feasibility non-randomised clinical trial
Source: Pilot Feasibility Stud. 2024 Feb 21;10:37. doi: 10.1186/s40814-024-01446-y (PMC10880214; doi:10.1186/s40814-024-01446-y)
Supplement: Supplementary file 2 — Additional file 2: Appendix 2. Sample INTERACTION intervention programs. [file 40814_2024_1446_MOESM2_ESM.docx]

Appendix 2

Sample INTERACTION intervention programs

**THEME: SPORTS**

| **PHYSICAL ACTIVITES**  Sports game including:   - egg and spoon race - 10 pin bowling - Throw bean bags in the hole   **Anticipated target in the older adults?**  Physical activity – Walking, sit to stand, balancing, coordination, strength    **Anticipated target in the children?**  Social skills, movement, coordination | **COGNITIVE ACTIVITIES**   - Show and tell by Denise (kids asking questions) - Matching sports things (e.g. pool and swimming goggles) - Song/dance with actions and movements     **Anticipated target in the older adults?**  Memory, rrecalling  Remembering dance moves    **Anticipated target in the children?**  Thinking skills, asking questions, rremembering dance moves | **SOCIAL ACTVITIES**   - Paired activities - Sport themed puzzle - Coloring in (sport theme) - Singing together     **Anticipated target in the older adults?**  Relationship building    **Anticipated target in the children?**  Turn-taking, social skills, relationship building, physical with dancing |
| --- | --- | --- |

**THEME: PARTY**

| **PHYSICAL ACTIVITES**   - Dancing to music - Creating and decorating the room - Party games e.g. Musical Chairs - Pass the Parcel     **Anticipated target in the older adults?**  Balance, sit to stand, walking, fine motor skills with decorating. Increase time in standing and reduce breaks in chairs for dancing and games    **Anticipated target in the children?**  Coordination, fine motor skills | **COGNITIVE ACTIVITIES**   - Welcome song - Memory/ Story building Game - Dancing to music with fixed steps - Remember to dress in party outfit - Story telling by older adults via show and tell     **Anticipated target in the older adults?**  Memory training  Some cognition needed for musical chairs also –processing speed.    **Anticipated target in the children?**  Concentration on instructions | **SOCIAL ACTVITIES**   - Discussion over pizza lunch - Knowledge sharing     **Anticipated target in the older adults?**  Improve mood, engagement, fun    **Anticipated target in the children?**  Empathy, engagement, fun |
| --- | --- | --- |
